# Acute Lymphoblastic Leukemia with Hypereosinophilia in a Child: Case Report and Literature Review

**DOI:** 10.3390/ijerph15061169

**Published:** 2018-06-04

**Authors:** Valentina Ferruzzi, Elisa Santi, Grazia Gurdo, Francesco Arcioni, Maurizio Caniglia, Susanna Esposito

**Affiliations:** 1 Pediatric Clinic, Department of Surgical and Biomedical Sciences, Università degli Studi di Perugia, Piazza Menghini 1, 06132 Perugia, Italy; ferruvale@libero.it (V.F.); elisa.santi1988@gmail.com (E.S.); 2 Pediatric Oncohematology Unit, Azienda Ospedaliera, Piazza Menghini 1, 06132 Perugia, Italy; grazia.gurdo@ospedale.perugia.it (G.G.); francesco.arcioni@ospedale.perugia.it (F.A.); maurizio.caniglia@alice.it (M.C.)

**Keywords:** acute lymphoblastic leukemia, blasts, bone marrow, hypereosinophilia, leukemia

## Abstract

**Background**: Hypereosinophilia in children can be primary or secondary. Numerous malignant diseases can cause hypereosinophilia, but it is seldom caused by acute lymphoblastic leukemia (ALL). In the event of protracted hypereosinophilia, it is extremely important to make a correct differential diagnosis. **Case presentation**: We present the case of an 11-year-old boy of Moroccan origin with ALL with hypereosinophilic onset (eosinophils in peripheral blood, 10,000/µL) in the absence of other signs of neoplastic disease, and compare this case with 61 similar cases in the literature. Following hospital admission, the patient initially presented with headache-caused nocturnal awakenings, evening fever, and cough, and he also lost approximately 7 kg in weight in a month not associated with sweating or itching. We first performed bone marrow aspiration, which showed an increase in eosinophils without cellular morphological abnormalities, and bone marrow immunophenotyping showed that 4.5% of cells had a phenotype compatible with lymphoid blasts. A lumbar puncture was negative. Given the poor marrow involvement, it was necessary to repeat a new bone marrow aspiration two days later, which showed an increase in blasts to 14%. A concomitant bone marrow biopsy showed an infiltration of blasts typical of B-cell ALL equal to 20–30% with associated hypereosinophilia. Cytogenetic analysis showed an hyperdiploid karyotype: 53–55, XY, +X, add(1)(q21q25), +4, +9, +10, +14, +2, +1, +21/46, XY. **Conclusions**: ALL is one of the possible causes of persistent hypereosinophilia. In patients with ALL and hypereosinophilia, peripheral hypereosinophilia can precede the appearance of blasts. Due to the negative prognosis and the increased risk of complications in these patients, bone marrow aspiration and biopsy are recommended if common causes of secondary hypereosinophilia are excluded.

## 1. Background

Hypereosinophilia (HE) is a relatively common condition. It is diagnosed when the eosinophil count is >500/µL, and depending on the eosinophil count in the peripheral blood, it may be graded as mild (500–1500/µL), moderate (1500–5000/µL), or severe (>5000/µL) [1,2]. Both primary (clonal) and secondary (reactive) forms have been described. Primary HE is usually associated with clonal abnormalities of myeloid lineage, whereas reactive HE accompanies several diseases in which T-cell-dependent cytokine production is the base of HE. Among the conditions that cause secondary HE, there are infections (by different pathogens, including parasites), atopy and allergy, skin and lung diseases, kidney diseases, autoimmune diseases, immunodeficiencies (i.e., Wiskott–Aldrich syndrome, Job syndrome, Nezelof syndrome, graft-versus-host disease, IgA deficiency), systemic mastocytosis, abnormal release of cytokines by immunophenotypically aberrant T lymphocytes, and neoplastic diseases. In less than 1% of the cases, reactive HE is the consequence of acute lymphoblastic leukemia (ALL) [1,3].

Early diagnosis of ALL is essential to assure adequate and effective therapy. Unfortunately, HE frequently precedes common signs and symptoms of ALL by several weeks or months [4,5]. Moreover, in several cases, at onset, disease manifestations are different from those usually detected in ALL and include signs and symptoms caused by eosinophil infiltration of body organs and systems. Diagnosis can be difficult and delayed with high risk for the patient. Here, a case of HE in a child with ALL is reported. Moreover, clinical manifestations of this case are compared to those of the patients aged less than 25 years of age with HE and ALL described and reported in PubMed up until 31 December 2017. This report can provide useful information for understanding the pathophysiological mechanism of ALL associated with HE and for managing HE-related clinical symptoms, favoring a prompt diagnosis of a severe but treatable disease.

## 2. Case Presentation

An 11-year-old boy of Moroccan origin was admitted to the Pediatric Ward of the Perugia General Hospital in July 2016 because, in the last 15 days, he had suffered from mild fever, chills and night sweats, severe itching, continuous dry cough, and right temporal headache. Moreover, in the month before hospital admission, he had lost approximately 7 kg in weight. Finally, a previous evaluation of blood cell count had revealed HE (eosinophils 10,000/µL) without other significant alterations. The patient denied having recently taken drugs and had had contact with animals. His last trip to his country of origin was in December 2015.

On admission, a blood cell count and a morphological evaluation of a peripheral blood smear confirmed HE (white blood cells 21,950/µL, with eosinophils 13,762/µL, hemoglobin 11.9 g/dL, and platelets 237,000/µL) the absence of other cellular morphological abnormalities. In the following days, the patient presented fatigue and worsening of headache; therefore, attempts to evaluate HE and other signs and symptoms origin were made. A parasitological fecal examination and a Scotch tape test were performed on three samples with negative results. Organ infiltration was excluded through chest X-ray, brain magnetic resonance imaging, echocardiography, and abdominal ultrasound. Toxoplasma, *Plasmodium falciparum*, *Leishmania*, *Schistosoma*, *Echinococcus*, viral hepatitis, and HIV infection were also excluded. Results of tests regarding comprehensive metabolic panel, inflammatory markers, and autoantibodies were negative excluding autoimmune diseases.

**Figure 1:**
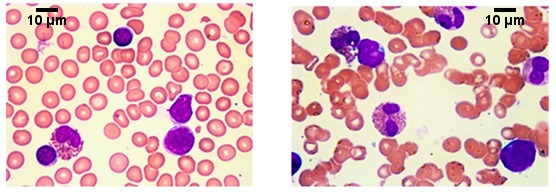
Bone marrow smear showing an infiltration of blasts typical of B-cell ALL equal to 20–30% with associated hypereosinophilia.

A bone marrow aspiration was performed approximately 20 days after hospital admission. Morphological examination documented an increase in eosinophils without cellular morphological abnormalities, and bone marrow immunophenotyping showed that 4.5% of the cells had a phenotype compatible with lymphoid blasts. A lumbar puncture was negative. However, given the poor marrow involvement, it was necessary to repeat the bone marrow aspiration two days later; the results of the new aspiration showed an increase in blasts to 14% (Figure 1) with a normal immunophenotype on mature B ant T cells. Concomitant bone marrow biopsy showed a cellularity of 40% with an infiltration of blasts typical of B-cell ALL equal to 20–30% with marked associated hypereosinophilia.

Cytogenetic analysis showed a hyperdiploid karyotype: 53–55, XY, +X, add(1)(q21q25), +4, +9, +10, +14, +2, +1, +21/46, XY; FISH analysis confirmed the numeric and structural abnormalities revealed by karyotype, without cryptic additional events. In particular, no abnormalities were found on chromosome 5. The main molecular leukemic rearrangements—including BCR/ABL, MLL/AF4, TEL/AML1, and E2A/PBX1—were negative. The in vitro production of interleukin (IL)-5 by T CD4 + lymphocytes was increased (41%) compared to the negative control (11%).

A few days after the start of prednisone prophase, a marked drop in peripheral eosinophil count and rapid clinical improvement were evident. On day 78, the patient was assigned to the standard risk group of the AIEOP ALL 2009 protocol. The patient is now undergoing maintenance therapy (6 Mercaptopurin plus Methotrexate) and will stop it on August 2018. Until now, the patient remains in complete remission; no further increase of eosinophil count was demonstrated.

## 3. Discussion

According to the algorithm suggested by the revised 2016 World Health Organization (WHO) Classification of Eosinophilic Disorders, the first step that should be made to classify a HE case is the screening of secondary cases [1]. Medical history, clinical signs and symptoms, and laboratory and imaging tests can significantly contribute to the identification of the condition that causes HE and favor risk stratification and management. When reactive causes of HE are excluded, the following step is the evaluation of primary bone marrow disorders. Blood smear, blood tests, and bone marrow morphologic, cytogenetic, and immunophenotypic evaluation can lead to the diagnosis of one of the myeloid neoplasms recently classified by WHO on the base of clinical manifestations and genetic characteristics (acute myeloid leukemia and related neoplasms, myeloproliferative neoplasms, myelodysplastic syndromes, myelodysplastic/myeloproliferative neoplasms, mastocytosis, and myeloid/lymphoid neoplasms) [1]. Moreover, in a lower number of cases, a lymphoid variant of HE (T-cell lymphomas, Hodgkin lymphoma, and ALL) can be diagnosed. Evidence of ALL makes the screening of the peripheral blood superfluous for genetic alterations such as *FIP1L1-PDGFRA* gene fusion, reciprocal translocations involving 4q12 (*PDGFRA*), 5q31-q33 (*PDGFRB*), 8p11–12 (*FGFR1*), or 9p24 (*JAK2*) that are recommended by WHO [1] and are useful to decide risk and risk-adapted therapy for myeloid neoplasms. On the contrary, it should be mandatory to perform cytogenetic analysis of lymphocytes in order to evaluate the most common abnormalities that can explain HE pathogenesis and characterize severity and response to therapy of ALL.

The mechanism of HE that accompanies some neoplastic diseases has not yet been fully clarified. It has been suggested that antigens expressed by tumor cells or exogenous factors such as viral infections can lead to T-cell stimulation and increased production of eosinophilopoietic growth factors [1]. However, regarding the development of HE in patients with ALL it seems likely that it is the consequence of a mixture of clonal and reactive processes [6]. Clonal origin of HE can be excluded as precursors of eosinophils in the bone marrow of these individuals have no genetic abnormalities suggesting that these cells have different hematopoietic origin. However, abnormal T-cell lymphocytes can be detected in lymphoid variant of HE syndromes. Absence of CD3 (e.g., CD3^−^CD4^+^), presence of immature T-cell (e.g., CD3^+^CD4^−^CD8^−−^), elevated CD5 expression on CD3-CD4^+^ cells, and loss of surface CD7 and/or expression of CD27 have been repeatedly described in these patients [7]. It has been supposed that these abnormal lymphocytes can produce significant amounts of Th2 cytokines (IL-3, IL-4, IL-5, IL-13) capable of inducing eosinophil proliferation and survival. This hypothesis is strongly supported by the evidence that in many of patients with HE and ALL, a relevant increase in blood concentration of one or more of these cytokines can be demonstrated. Further evidence of the strict relationship between HE and Th2 cytokine production is given by the few patients with ALL-associated HE in whom genetic abnormalities, such as t (5; 14) (q31; q32) and del (5) (q15q33), induce IL-3 gene activation and relevant production of this eosinophilopoietic cytokine [8,9,10].

Cases of HE secondary to ALL are very rare. In addition to this child, only 61 patients <25 years (mean age 11 years) with HE and ALL have been described since 1973 to December 2017 (Table 1) [8,9,10,11,12,13,14,15,16,17,18,19,20,21,22,23,24,25,26,27,28,29,30,31,32,33,34,35,36,37,38,39,40,41,42,43,44,45,46,47,48,49,50,51,52,53,54,55,56,57,58].

Most of them had clinical and laboratory characteristics that can be found in the described patient and can be considered typical of this kind of disease. Generally, ALL with HE is a B-cell disease that occurs mainly in males. Frequently, HE is diagnosed before the onset of classic signs and symptoms of ALL and disappears after therapy during remission of the disease. Moreover, in many patients, no blast is detected in the peripheral smear when HE is diagnosed. Finally, several clinical manifestations not found in typical ALL cases accompany HE. They are mainly due to eosinophil infiltration of different body organs and systems and can significantly condition final prognosis of ALL independently of the blast level at onset [33]. In this case, in addition to non-specific symptoms, such as fever and fatigue, respiratory symptoms were clinically important, as in 43% of reported patients. In other cases, HE was associated with skin erythema (33%; often with a vasculitis component) and cardiovascular alterations (13%). Particularly, the cardiac infiltration of eosinophils can cause a progression towards restrictive cardiomyopathy, leading to Loeffler endocarditis and death [22,33,59]. Moreover, an increased risk of thromboembolic events was found, sometimes as early as the onset of the disease, and this must be completely taken into account by clinicians because therapy of ALL involves protocols that include prothrombotic drugs such as L-asparaginase [7,60,61]. In this case, increase in IL-5 blood concentration was found, confirming the reactive origin of HE. No genetic abnormality of lymphocytes was found.

## 4. Conclusions

As our case and review of the literature showed, ALL is one of the possible causes of persistent hypereosinophilia. In patients with ALL and hypereosinophilia, peripheral hypereosinophilia can precede the appearance of blasts. Due to the negative prognosis and the increased risk of complications in these patients, bone marrow aspiration and biopsy are recommended if common causes of secondary hypereosinophilia are excluded.

## Figures and Tables

**Table 1:** Characteristics of the cases reported in the literature from 1973–present of acute lymphoblastic leukemia (ALL) with hypereosinophilic onset in patients younger than 25 years.

Data	Characteristic (*n* = 61)
Gender, male (%)	34 (55.7%)
Age, median years (range)	10 (1–25)
Main clinical characteristics (%)	Fever (62.3%), respiratory symptoms (42.6%), organomegaly (31.1%), osteoarticular pain (29.5%), rash (29.5%), asthenia (24.6%), lymphoadenopathy (18%), cardiovascular symptoms (9.8%)
First blood cell count	
White blood count, median cells/µL (range)	48,300 (15,000–218,000)
Eosinophil count, median cells/µL (range)	30,000 (270–135,000)
Platelets, median cells/µL (range)	121,000 (100,000–440,000)
Hemoglobin, median value in g/dL (range)	10.5 (4.7–14.2)
Blasts, median % (range)	Present in 16 cases (26.3%): 7% (3–90%)
Diagnosis	
B-cell ALL, *n*. (%)	29 (47.5%)
Pre-B ALL, *n*. (%)	12 (19.7%)
Common ALL, *n*. (%)	5 (8.0%)
Other, *n*. (%)	15 (24.8%)
Cytogenetics	
46 XX, *n*. (%)	18 (29.5%)
Other forms, *n*. (%)	t (5; 14) 8 (13%), iperdiploid 8 (13%), del (5) 2 (3.3%), other 4 (6.7%)
Not available, *n*. (%)	21 (34.5%)
Outcome	
Complete resolution, *n*. (%)	24 (39.3%)
Complete resolution after bone marrow transplantation, *n*. (%)	3 (5%)
Death, *n*. (%)	22 (36%)
